# Acoustic Overexposure Increases the Expression of VGLUT-2 Mediated Projections from the Lateral Vestibular Nucleus to the Dorsal Cochlear Nucleus

**DOI:** 10.1371/journal.pone.0035955

**Published:** 2012-05-03

**Authors:** Matthew Barker, Hans Jürgen Solinski, Haruka Hashimoto, Thomas Tagoe, Nadia Pilati, Martine Hamann

**Affiliations:** Department of Cell Physiology and Pharmacology, Leicester University, Leicester, United Kingdom; Tokyo Medical and Dental University, Japan

## Abstract

The dorsal cochlear nucleus (DCN) is a first relay of the central auditory system as well as a site for integration of multimodal information. Vesicular glutamate transporters VGLUT-1 and VGLUT-2 selectively package glutamate into synaptic vesicles and are found to have different patterns of organization in the DCN. Whereas auditory nerve fibers predominantly co-label with VGLUT-1, somatosensory inputs predominantly co-label with VGLUT-2. Here, we used retrograde and anterograde transport of fluorescent conjugated dextran amine (DA) to demonstrate that the lateral vestibular nucleus (LVN) exhibits ipsilateral projections to both fusiform and deep layers of the rat DCN. Stimulating the LVN induced glutamatergic synaptic currents in fusiform cells and granule cell interneurones. We combined the dextran amine neuronal tracing method with immunohistochemistry and showed that labeled projections from the LVN are co-labeled with VGLUT-2 by contrast to VGLUT-1. Wistar rats were exposed to a loud single tone (15 kHz, 110 dB SPL) for 6 hours. Five days after acoustic overexposure, the level of expression of VGLUT-1 in the DCN was decreased whereas the level of expression of VGLUT-2 in the DCN was increased including terminals originating from the LVN. VGLUT-2 mediated projections from the LVN to the DCN are likely to play a role in the head position in response to sound. Amplification of VGLUT-2 expression after acoustic overexposure could be a compensatory mechanism from vestibular inputs in response to hearing loss and to a decrease of VGLUT-1 expression from auditory nerve fibers.

## Introduction

The dorsal cochlear nucleus (DCN) in the auditory brainstem is a major termination point of the auditory nerve and auditory nuclei [1]–[6]. Principal DCN fusiform cells and granule interneurons compose a circuit finely tuned to encoding spectral components of sound [7], [8] and receive direct and indirect acoustic inputs from the auditory nerve [5], the ventral cochlear nucleus [2]–[4] and olivary and peri-olivary regions of the auditory brainstem [1], [6]. The DCN also plays an important role in the integration of non auditory inputs from sensory locations [9]. DCN granule cells and their parallel fiber axons are a site of integration of multimodal sensory inputs such as those from the trigeminal ganglion [10], the spinal trigeminal nucleus [11]–[13], the pontine nucleus [14], the cuneate nucleus and gracile nuclei [13], [15]–[18] and the raphe nucleus [19] possibly encoding proprioceptive information on the position of the ears relative to the sound source [20] or suppressing body-generated sounds or vocal feedback [9], [21], [22]. Although multisensory integration also includes projections from primary and secondary vestibular afferent fibers to the DCN [23], [24], the nature of synaptic projections from vestibular nuclei to the DCN remains unidentified.

Synaptic projections can be specifically associated with vesicular glutamate transporters that package glutamate into synaptic vesicles [25]–[27]. Both VGLUT-1 and VGLUT-2 [25], [28] but not VGLUT-3 are expressed in terminals in the rodent cochlear nucleus [29] although VGLUT-3 has been identified in somata in the cochlear nucleus [30]. In the DCN, VGLUT-1 and VGLUT-2 are distinctly associated with synaptic terminals of the auditory nerve and multisensory projections, respectively [31]. Type I auditory nerve fiber terminals co-label with VGLUT-1 and not VGLUT-2 [31] whereas projections originating from the spinal trigeminal nucleus, the cuneate nucleus and the lateral reticular nucleus co-label with VGLUT-2 and not VGLUT-1 [12], [31].

The vestibular nuclear complex lies in the floor of the fourth ventricle and consists of four major nuclei, namely the medial, lateral, superior and inferior vestibular nuclei. The vestibular nuclear complex controls eye movements, reflex postural head and neck, movements and balance during stance and gait as well as modulation and modification of autonomic function to maintain homeostasis during changes in body posture [32]. Both VGLUT-1 and VGLUT-2 are widely expressed in all sub-nuclei of the vestibular nucleus complex where the axon terminals are either VGLUT-1 single labelled, VGLUT-2 single labelled or VGLUT-1, VGLUT-2 double labelled [33]. Here we tested whether vestibular inputs project to the DCN and whether vestibular projections to the DCN specifically co-label with VGLUT-1 or VGLUT-2. Small boutons and larger irregular mossy fiber terminals in the DCN have been found to originate from multisensory inputs such as those from the cuneate and the spinal trigeminal nuclei [13], [31]. We studied whether LVN projections to the DCN also terminate into boutons and mossy fiber terminals. Vestibular projections to the DCN may well provide information on head orientation with respect to the DCN, enabling the encoding of spectral components of sound [21] and therefore playing a complementary role in the orientation of the head to locate sound.

Previous studies have shown that cochlear kanamycin injections which produce deafness, resulted in a significant reduction of VGLUT-1 in the regions of the ventral and dorsal cochlear nucleus receiving auditory nerve inputs as well as an increase of VGLUT-2 in regions receiving non auditory inputs [34]. Deafness can also be induced by acoustic overexposure (AOE) and is associated with degeneration of the auditory nerve axonal endings in the cochlear nucleus [35]–[37]. We therefore examined whether the differential distribution of VGLUT-1 and VGLUT-2 is affected after exposure to loud sound and whether the expression of VGLUTs associated to a specific vestibular projection to the DCN is changed after AOE.

Here we characterised VGLUT-2 mediated projections from the lateral vestibular nucleus (LVN) to the DCN by combining dextran amine neuronal tracing method with immunohistochemistry. We also showed that exposure to loud sound resulted in an increase in the number of VGLUT-2 positive terminals originating from the LVN and discuss its role within the context of cross modal compensation of hearing deficit.

## Materials and Methods

Experiments were carried out in accordance with the UK Animals (Scientific Procedures) Act 1986 and approved by the Home Office and Leicester University's Ethical Committee (PIL 80/8158, PPL 80/1660).

### Dissection

Forty six Wistar and Lister Hooded rats P18 to P27 day-old were culled by decapitation and their brains were immediately transferred to ice-cold low sodium artificial solution containing (in mM): 250 sucrose, 2.5 KCl, 10 glucose, 1.25 NaH_2_PO_4_, 26 NaHCO_3_, 0.5 ascorbic acid, 0.1 CaCl_2_ and 4 MgCl_2_ (pH 7.4 when gassed with 95% O_2_, 5% CO_2_). The brainstem was dissected out of the brain, oriented in its coronal or its sagittal plane and glued onto a mounting plate that was placed in a slicing chamber of a VT1000S vibroslicer (Leica Microsystems Ltd, Milton Keynes, UK) containing the same ice cold solution as described above. Axonal tracing was performed on the isolated brainstem as described below.

### Axonal tracing

One millimetre thick slices were cut under binocular control using a MZ75 stereomicroscope (Leica Microsystems Ltd). Slices were cut from the top of the brainstem until the DCN was reached. Injections were subsequently performed below the tissue surface to a depth of 500 µm ensuring that subsequent sections contain both the LVN and the DCN (32 animals used in total for tracer injections) [38]. Extracellular medium was then changed to an artificial cerebrospinal solution containing (in mM) 125 NaCl, 2.5 KCl, 10 glucose, 1.25 NaH_2_PO_4_, 26 NaHCO_3_, 2 sodium pyruvate, 3 myo-inositol, 0.5 ascorbic acid, 2 CaCl_2_, and 1 MgCl_2_ (pH 7.4 when gassed with 95% O_2_ ∶ 5% CO_2_). Dextran amine dye (see below for composition) was delivered under binocular control, using a custom made combined pressure and electroporation system coupled to a double-barrelled glass pipette (TGC200-10, Clark Electromedical, Harvard Apparatus Ltd, Kent, UK) as described in Barker *et al.* [39] (Figure S1). Five ms square wave pulses were applied at 100 Hz for up to 10 s with 2 pressure ejection pulses of 10–20 psi, 10 ms occurring mid-way through the electroporation. The total electrode diameter was about 150 µm and allowed localised delivery of dextran amine dye within areas up to 200 µm diameter in the DCN or the LVN. Fluorescent dextran tetramethylrhodamine (fluoro-ruby) 10,000 MW (Invitrogen) was made up as a 10% stock solution in 0.4 M KCl and further diluted at 1∶10 in a solution containing (in mM): 150 NaCl, 10 HEPES, 2.5 KCl, 11 glucose, 1 MgCl_2_ and 2.5 CaCl_2_ at pH 7.3. Axonal tracing was performed on brainstems extracted from control animals and 5 days after initial exposure to loud sound using age matched rats from the same litters.

### Incubation and slicing

Brainstems attached to their mounting plates were then transferred to an incubation chamber containing the artificial cerebrospinal solution detailed above and bubbled with 95% O_2_∶ 5% CO_2_ at 37°C for a period of 3 to 4 hours. After the incubation period, brainstems were either frozen down for immunohistochemistry (see below) or sliced before final observation. Slicing was performed by transferring the mounting plate with the glued brainstem to the vibroslicer slicing chamber and 120 µm thick slices were cut in the ice-cold low sodium artificial solution described above. Slices were performed up to a depth of 600 µm which contain both the DCN and the LVN. Slices were fixed in 4% paraformaldehyde for 10 minutes before being washed in 1× PBS for 10 minutes. Slices were finally mounted on slides with 1% agarose and viewed with an Olympus Fluoview FV300 confocal microscope (Olympus Ltd, Watford, UK) for final observation.

### Serial section

Axonal tracing was performed on the isolated brainstem as described above. Serial sections were taken in slices containing the DCN and LVN (in both coronal and sagittal planes). Transmitted light images were taken using a Nikon eclipse TE2000 microscope and fluorescently labelled cells were imaged using an Olympus Fluoview FV300 confocal microscope. Transmitted light images were then overlaid and the outlines of the sections including the DCN were traced over using Powerpoint. Labelled cell bodies within the LVN were also overlaid on the reconstruction and then represented by red dots with each dot representing a cell body.

### Immunohistochemistry

Immunohistochemistry was performed on tissue extracted from control animals (see below) and 5 days after initial exposure to loud sound using age matched rats from the same litters. Brainstems were rapidly frozen in Tissue Tek (Sakura) after their incubation with dextran amine dye, using hexane and dry ice. Frozen tissue was processed within a month and sectioned at 20 µm using a cryostat. Slices were mounted on Polysine coated slides and fixed in 4% paraformaldehyde for 10 minutes. Slides were washed for 3×5 min in 1× Dullbeco's PBS (Invitrogen) with 0.1% Triton X-100 (PBS-T solution). After washing, sections were incubated with 1% bovine serum albumin and 1% goat serum in 1× PBS with 0.1% Triton X-100 (blocking buffer) for 1 hour at 20°C. Sections were then incubated with anti-VGLUT antibodies (VGLUT-1 and VGLUT-2, 1∶1000 and 1∶2000 dilution respectively from Synaptic Systems, Catalogue numbers, VGLUT-1: 135–304., VGLUT-2: 135–402) in blocking buffer overnight at 4°C. Slides were then washed with PBS-T for 6×10 min. The secondary antibody (goat anti-rabbit Alexa-fluor488 or goat anti-guinea pig Alexa-fluor568 Molecular Probes, 1∶1000) was applied for 2 h at 20°C. Sections were washed in PBS-T solution for 6×10 min and then mounted with Prolong Gold antifade reagent (Molecular Probes).

### Imaging and analysis

Images were obtained using an Olympus Fluoview FV300 confocal microscope (Olympus Ltd). When imaging VGLUT immunoreactivity, confocal settings were determined using samples from animals aged P25 and the settings remained constant for all other imaging. To quantify VGLUT immunoreactivity sections were selected from an even distribution throughout the cochlear nucleus (a section from each of the 25^th^, 50^th^ and 75^th^ percentile for each animal). The images were converted to 8 bit TIFF files in ImageJ (NIH software). Analysis was conducted as described in [34]. Analysis was performed on regions that receive primarily type 1 auditory nerve inputs (the magnocellular cellular area of the VCN [31], the deep layer and the molecular layer of the DCN). Analysis was also performed on regions receiving primarily non auditory inputs that included the shell region, the fusiform cell layer of the DCN and the deep layer of the DCN [11]. The whole region mentioned above was selected per section using the freehand draw tool in ImageJ with the selection areas being identical for both VGLUT-1 and VGLUT-2. Assessment of intensity was performed by using the measure function in ImageJ. Size of cell bodies in the LVN and terminals in the DCN were measured using ImageJ. To analyze puncta density, a 20 µm×20 µm grid was placed over photomicrographs from each area of the cochlear nucleus and the number of puncta counted. For cell number comparisons, the number of whole cell bodies per entire captured field of view (55000 µm^2^ using a 60× objective) were counted for alternate coronal and sagittal sections. The labelled terminals were classified into two different subtypes, mossy fiber like terminals which have large irregular endings (≥2 µm) and small boutons (<2 µm) [31].

### Patch clamp recording and stimulation

Experiments were conducted in P18–25 day old rats as the use of juvenile rats allowed reliable and stable whole cell recordings. Patch clamp recordings were performed in control animals (see below) and 5 days after initial exposure to loud sound using age matched rats from the same litters. The dissection of the brainstem from 14 Wistar rats was performed as described above. Sagittal slices (160–180 µm thick) containing the LVN and the DCN were cut in low sodium artificial cerebrospinal solution before being transferred to the experimental chamber containing the artificial cerebrospinal solution (both solutions are described above). DCN cells were visualised with differential interference contrast (DIC) optics on an Axioskop microscope (Zeiss, UK) with a 40× N.A. 0.75 water-immersion lens. Whole-cell patch-clamp recordings were made from fusiform and granule cells using thick-walled glass pipettes (GC150F-7.5 Clark Electromedical, Harvard Apparatus, Ltd Kent, UK) with a Multiclamp 700A amplifier (Axon Instruments, Foster City, CA, USA), connected to an analogue to digital converter (Digidata 1322A, Axon Instruments) filtered at 6 kHz (8-pole Bessel filter) and sampled at 20 kHz. Currents were recorded with pCLAMP 9.2 software (Axon Instruments). Cells were maintained at a holding potential of −70 mV and whole-cell access resistances of less than 10 MΩ were not compensated. The intracellular solution contained 0.1% lucifer yellow and (in mM) 97.5 K gluconate; 32.5 KCl; 5 EGTA; 10 HEPES; 1 MgCl_2_; 2 NaCl (pH of 7.2 with KOH). Excitatory postsynaptic currents were elicited by stimulating the lateral vestibular nucleus with a concentric bipolar electrode (FHC Inc, Bowdoinham, ME, USA) consisting of a platinum iridium inner pole of 50 µm and a stainless style outer diameter of 200 µm. Electrical pulses of 30–80 V (0.5–2 mA) and 0.1 ms duration were provided by a Digitimer DS2 A isolated stimulator (Digitimer Ltd, Welwyn Garden City, UK) triggered by the pCLAMP software. Pharmacological blockers were bath applied using a peristaltic pump (Gilson Minipuls 3). Excitatory post synaptic currents were recorded in the presence of strychnine (10 µm) and gabazine (20 µm) and were blocked by NBQX disodium salt (10 µm, 2,3-dihydroxy-6-nitro-7-sulfamoyl-benzo-(F)-quinoxalin) and D-AP5 (50 µm, D-2-amino-5-phosphonopentanoate). NBQX and D-AP5 were from Ascent Scientific (Weston-super-Mare, U.K.), gabazine was from Tocris Bioscience (Bristol, U.K.). All the other drugs used in this study came from Sigma Aldrich (Gillingham, U.K.). Once the whole cell recording was terminated, the pipette was gently removed from the cell by positive pressure and slices were fixed in 4% paraformaldehyde for 4 hours before being washed in 1× PBS for 15 minutes. Slices were finally mounted onto slides with 1% agarose with the cover-slip sealed with nail polish to prevent dehydration. Slices were kept overnight at 4°C before final observation with the Olympus Fluoview FV300 confocal microscope.

### Acoustic overexposure and auditory brainstem response recordings

Wistar rats aged between 19 and 22 days were used (15 control and 15 acoustic over exposure) as this represents an age where the onset of hearing has already occurred [40]. Rats were anesthetised with an intra-peritoneal injection of fentanyl (0.15 mg/kg), fluanisone (5 mg/kg, VetaPharma Ltd) and Hypnovel (2.5 mg/kg, Roche). Animals were placed in an open field sound-insulated chamber (custom designed) containing a 600 W High Power Horn Tweeter, frequency range 2–20 kHz (Maplin, UK), delivering a single frequency tone (14.8 kHz) at 110 dB SPL to both ears. Two exposures were conducted in two 3 hour sessions over a period of 2 days. Control animals were similarly anesthetized but unexposed to sound.

Auditory brainstem response recordings were performed before AOE and 5 days after the first day of exposure to loud sound. Recordings were performed by inserting positive, negative and ground electrodes subcutaneously at the vertex, the mastoid and the back, respectively [41]. Auditory brainstem responses were evoked by calibrated tone pips at varying frequencies of 8, 12, 16, 24 and 30 kHz (1 ms rise and fall times, 5 ms duration, 3 ms plateau) generated in the free field at 10 Hz by a waveform generator (TGA 1230, 30 MHz, Thurlby Thandar Instruments, USA) and delivered via an acoustic driver (Bruel & Kjaer type 4192, Denmark). Responses were recorded by an amplifier (Medelec Sapphire 2A, Oxford Instruments, UK), filtered between 5 and 10 kHz and averaged with 100–800 sweeps using custom designed software (CAP, GSK). Tone pips were progressively attenuated in either 10 or 3 dB SPL increments from an initial intensity of 94 dB SPL using a digital attenuator (PA4, Tucker Davis Technology, USA). The hearing threshold was defined as the lowest intensity at which an auditory brainstem response at peak one and two could be discerned.

### Statistical analysis

Means ± SEM were calculated using n samples which represented either the number of fields, terminals or grids as indicated in the text. N represents the number of animals for each condition. Statistical comparisons were performed by Mann-Whitney tests when comparing VGLUT-1 and VGLUT-2 intensities within regions of the cochlear nucleus and when comparing the effect of AOE on both VGLUT-1 and VGLUT-2 intensities in those regions. Although the test is non-parametric, it does assume that the two distributions are similar in shape and shape distributions were previously assessed as follows. Measurements of florescent intensity were taken along each layer of the DCN (at points approximately 25%, 50% and 75% along the length of each layer dorsal to ventral), in the MCD and in the shell region. Data were then compared using a one way ANOVA with Tukeys post hoc test. For all regions there was no significant difference in fluorescent intensity between all points. Unpaired T tests were used when comparing the size of labelled cell bodies in the LVN and when comparing terminal sizes under control conditions and after AOE; the percentage of VGLUT-2 labelled terminals originating from the LVN, the EPSC amplitude in control and exposed conditions. Significance was assessed for P<0.05. Data are represented as Mean ± SEM.

## Results

### Projections from the lateral vestibular nucleus to the dorsal cochlear nucleus

**Figure 1:**
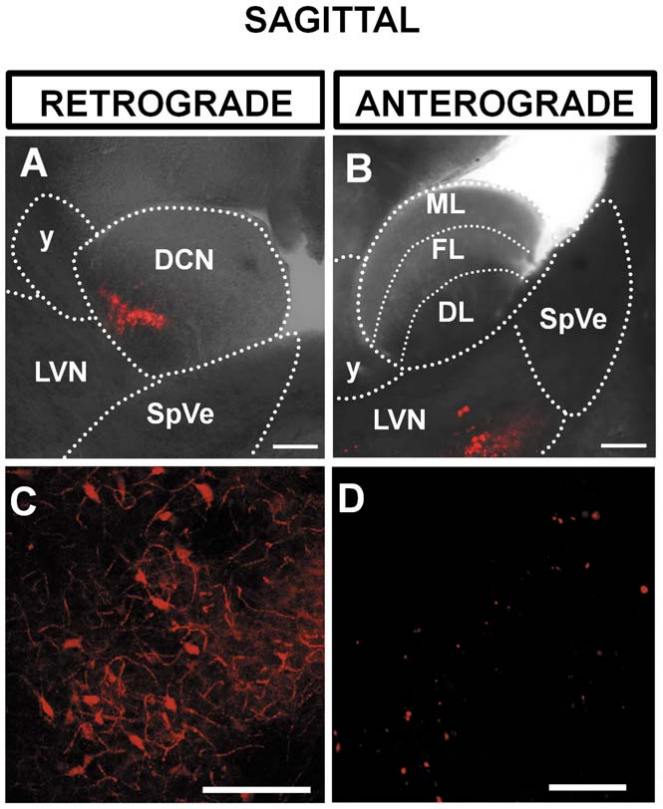
Sagittal brainstem slice showing a retrograde labelling of the lateral vestibular nucleus (LVN) following injection of dextran amine in the dorsal cochlear nucleus (DCN) (A,C) and an anterograde labelling of the DCN following injection of dextran amine in the LVN (B,D). (**A**) Overlay of a brightfield and fluorescence photomicrograph at 3 hours post injection of dextran amine showing the position of the LVN and the DCN relative to the spinal vestibular nucleus (SpVe) and the nucleus Y (y). The fluorescence in the DCN shows the injection site. (**B**) The LVN is labeled as a result of retrograde transport of dextran amine. (**C**) Overlay of a brightfield and fluorescence photomicrograph showing the injection site in the LVN. (**D**) Labeled terminals in the DCN as a result of anterograde transport of dextran amine. Scale bar: (A) and (B) 200 µm, (C) and (D) 20 µm. All slices are 120 µm thick. ML: molecular layer; FL: fusiform cell layer; DL: deep layer.

**Figure 2:**
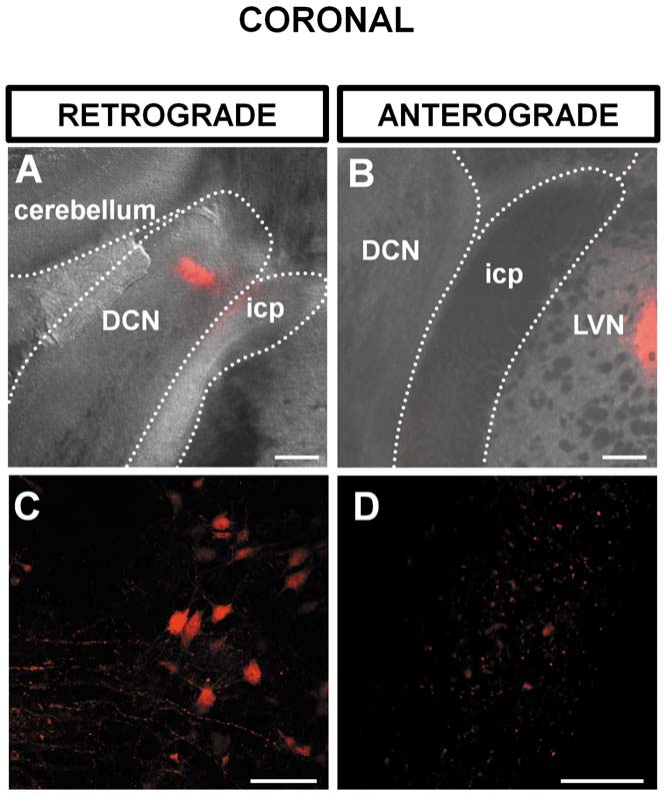
Coronal brainstem slice showing a retrograde labelling of LVN following injection of dextran amine in the DCN (A,C) and an anterograde labelling of the DCN following injection of dextran amine in the LVN (B,D). (**A**) Overlay of a brightfield and fluorescence photomicrograph at 3 hours post injection of dextran amine showing the position of the DCN relatively to the inferior cerebellar peduncle (icp) and the cerebellum. The fluorescence in the DCN shows the injection site. (**B**) The LVN is labeled as a result of retrograde transport of dextran amine. (**C**) Overlay of a brightfield and fluorescence photomicrograph showing the position of the dextran amine injection site in the LVN. (**D**) Labeled terminals in the DCN as a result of anterograde transport of dextran amine. Scale bar: (A) and (B) 200 µm, (C) and (D) 20 µm. All slices are 120 µm thick.

**Figure 3:**
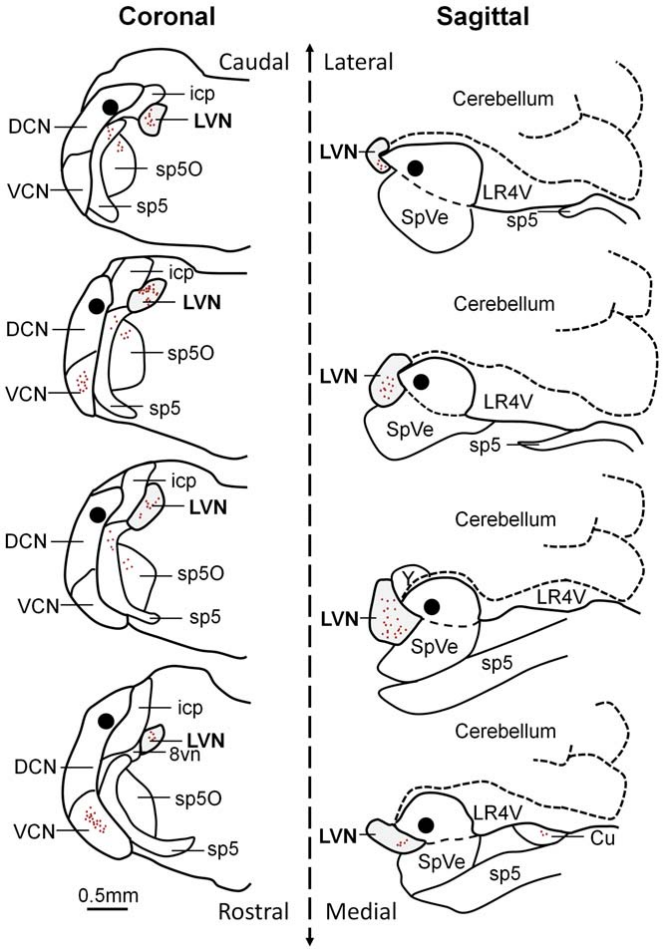
Reconstruction of retrograde cell body labelling in the LVN following an injection of dextran amine in the DCN. Injections were performed on coronal (left) and sagittal (right) brainstem preparations and serial sections of 100 µm were performed. The black area represents the injection site in the DCN and the red dots represent labelled cell bodies including those in the LVN (grey area). LVN: lateral vestibular nucleus; DCN: dorsal cochlear nucleus; VCN: ventral cochlear nucleus; icp: inferior cerebellar peduncle; sp5: spinal trigeminal tract; sp5O: spinal trigeminal nucleus; 8vn: vestibular route of 8^th^ nerve; SpVe: spinal vestibular nucleus; LR4V: lateral recess 4^th^ ventricle; Cu: cuneate nucleus.

The cochlear nucleus is divided into the ventral and the dorsal cochlear nucleus (DCN) [42], [43]. The DCN forms a tubercle lying below the cerebellum and consists of the molecular, the fusiform cell and the deep layer ([44], Figures 1B). Focal injections of dextran amine [39] in the DCN were confined to the fusiform and the deep layers of the DCN (Figure 1A). In each case labelled neurones were consistently observed in the ipsilateral lateral vestibular nucleus (ipsilateral LVN) whereas no labelling was observed in the contralateral LVN (N = 4). Figure 1A represents the position of the LVN and the DCN relative to the spinal trigeminal tract (sp5), spinal trigeminal nucleus (SpVe) the inferior cerebellar peduncle (icp) and the nucleus Y (y) in a sagittal section. Focal injection of dextran amine into the DCN resulted in labelled cell bodies in the LVN that were observed in sagittal sections (Figure 1C) and could also be observed in coronal sections (Figure 2B). This demonstrates retrograde transport of dextran amine from the DCN to the LVN as represented in Figure 3. Other nuclei previously documented were also observed and can be seen in representations of sagittal and coronal slices in Figure 3; these include projections from the ventral cochlear nucleus and the supra trigeminal nucleus [11], [31], [45]. The proportion of labelled cell bodies in coronal sections was about a third of the labelled cell bodies in sagittal sections (i.e. 31±15 n = 9 somata per field of view, N = 3 and 97±26, n = 9 somata per field of view, N = 3 respectively, P<0.01 unpaired t test). However, labelled cell bodies in the LVN were of similar diameters in sagittal sections (Figure 1C) and in coronal sections (Figure 2C) (17.0±1.8 µm, n = 263 terminals, N = 3 and 17.8±1.3 µm, n = 90 terminals, N = 3 respectively, P = 0.13 unpaired T test).

When dextran amine was injected into the LVN (Figure 1B, 2B) synaptic terminals were observed in the DCN (Figure 1D, 2D) demonstrating anterograde transport of dextran amine from the LVN to the DCN. Labelled terminals were found equally distributed between the DCN deep layer and fusiform layers (56% and 44% respectively with an average diameter of 1.7±0.4 µm, n = 355 terminals, N = 4) that was consistent with terminal diameters previously reported in the DCN [31].

### Functional synaptic projections from lateral vestibular nucleus to the dorsal cochlear nucleus

**Figure 4:**
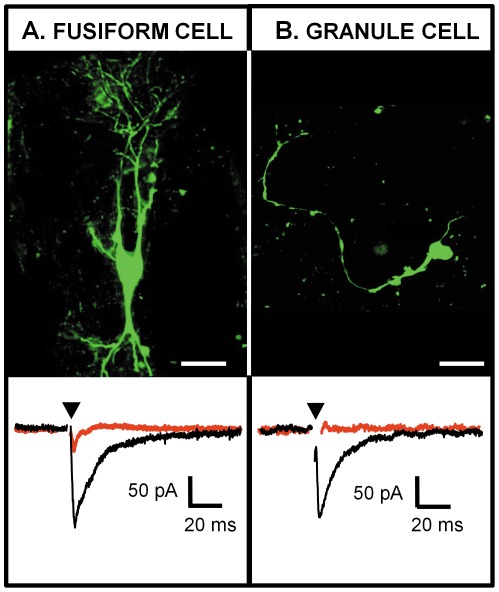
Glutamatergic post-synaptic currents (EPSCs) elicited in identified DCN cells by stimulating the LVN in a sagittal slice. (**A**) Photomicrograph of a DCN fusiform cell filled with lucifer yellow (top) and whole cell voltage clamp recording of this fusiform cell while stimulating the LVN (bottom). (**B**) Photomicrograph of a DCN granule cell filled with lucifer yellow (top) and whole cell voltage clamp recording of this granule cell while stimulating the LVN (bottom). Both cells were held at −68 mV and the LVN was stimulated at 0.3 Hz. Glutamatergic EPSCs are represented in black and are blocked by 50 µm D-AP5 and 10 µm NBQX (traces in red). Each trace represents an average of 10–20 single traces. The arrowhead represents the artifact of stimulus that has been removed for clarity. Scale bar: (A) 50 µm, (B) 20 µm.

To assess the presence of functional synaptic projections between the LVN and the DCN, whole cell recordings were performed in sagittal slices containing both nuclei. Seven fusiform cells (N = 6) were characterised by their location in the fusiform layer, their morphology (based on Lucifer yellow filling) and their passive properties (capacitance = 147±20 pF, membrane resistance = 113±19 MΩ, resting potential = −53±4 mV [46], [47]. Twelve granule cells (N = 5) were characterised by their location in the deep layer and by their passive properties (capacitance = 8±2 pF, membrane resistance = 1.4±0.2 GΩ, resting potential = −47±3 mV [48]. Once the whole cell recording was terminated, the morphologies of the cells were confirmed with confocal microscopy. Examples of fusiform and granule cells filled with lucifer yellow are shown in Figure 4A and B respectively. Stimulating the LVN induced excitatory post-synaptic currents in 3 out of 12 (25%) granule cells (example trace shown in Figure 4B) and 4 out of 15 (27%) fusiform cells (example trace shown in Figure 3A 4A). Synaptic currents of 170±20 pA in three granule cells and 48±14 pA in four fusiform cells were blocked by 50 µM D-AP5 and 10 µM NBQX indicating that synaptic transmission from the LVN to the DCN is mediated by glutamate release.

### Differential expression of VGLUT-1 and VGLUT-2

**Figure 5:**
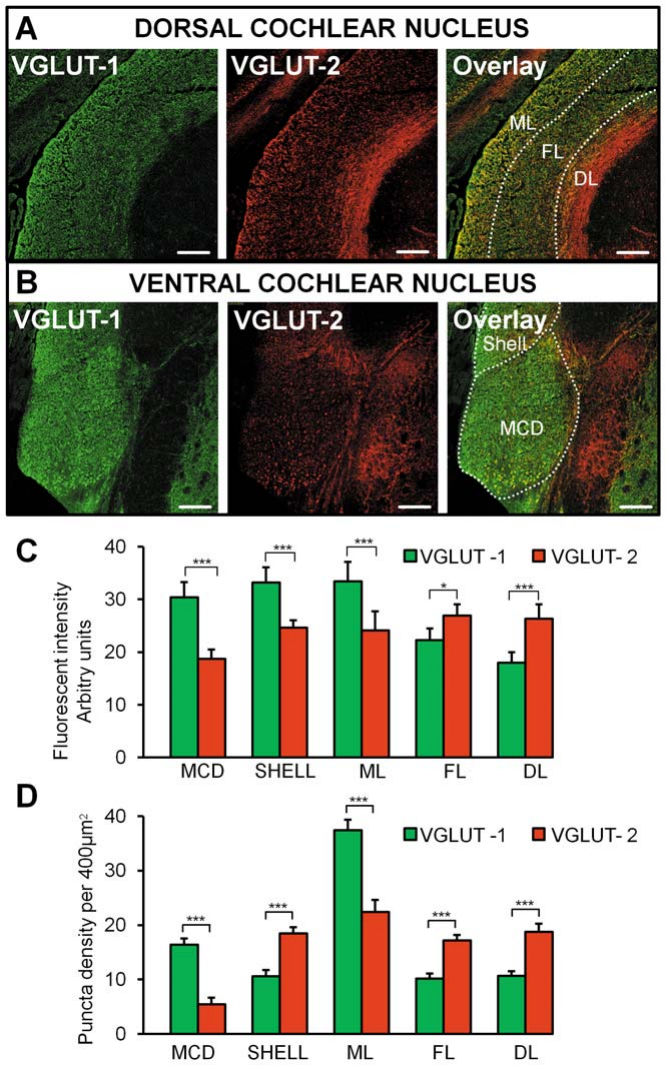
Expression of VGLUT-1 and VGLUT-2 in the DCN and the VCN. (**A**) Photomicrographs of VGLUT-1 and VGLUT-2 staining in the DCN with the layers being individually labelled (ML molecular layer, FL fusiform layer, DL deep layer). The overlay shows that VGLUT-1 is mainly present in the ML whereas VGLUT-2 is mainly present in the DL. (**C**) (**B**). Photomicrographs of VGLUT-1 and VGLUT-2 staining in the VCN. The overlay shows that VGLUT-1 is mainly expressed in the VCN in comparison to VGLUT-2. Scale bar: 200 µm. All slices are 20 µm thick. (**C**) Histograms representing the fluorescence intensity of VGLUT-1 and VGLUT-2 in the DCN layers, the MCD and the shell region. * p<0.05, *** P<0.001, NS non significant. (**D**) Histograms representing the puncta density of VGLUT-1 and VGLUT-2 labelled terminals in the DCN layers, the MCD and the shell region, *** P<0.001. ML: molecular layer; FL: fusiform cell layer; DL: deep layer.

VGLUT-1 intensity was strongest compared to VGLUT-2 in the VCN magnocellular domain (MCD), the shell region and the DCN molecular layer (p<0.001, Mann Whitney, n = 9 fields, N = 3, Figure 5 A–C). By contrast, the intensity of VGLUT-2 was higher in comparison to VGLUT-1 in the DCN fusiform layer and the deep layer (p<0.001, Mann Whitney, n = 9 fields, N = 3, Figure 5A and C). Our observations are consistent with a previous study performed in guinea pigs showing that VGLUT-1 and VGLUT-2 were mostly spatially segregated [31]. The puncta density was also analyzed (Figure 5D and Figure S2). Similar to the fluorescent intensity, VGLUT-1 puncta density was greatest in the MCD and DCN ML (p<0.001, Mann Whitney, n = 60 grids, N = 3, Figure 5A and D, Figure S2A and C). The density of VGLUT-2 was greatest in the shell region, the DCN fusiform layer and the deep layer (p<0.001, Mann Whitney, n = 60 grids, N = 3, Figure 5D and Figure S2B, D and E). The notable exception was in the shell region where the VGLUT-1 puncta density was smaller compared to VGLUT-2, whereas the fluorescent intensity was greater. This was due to larger and brighter VGLUT-1 in the shell region compared to VGLUT-2, therefore giving rise to a low puncta density but high fluorescent intensity.

### VGLUT-2 mediated projections from the lateral vestibular nucleus to the dorsal cochlear nucleus

**Figure 6:**
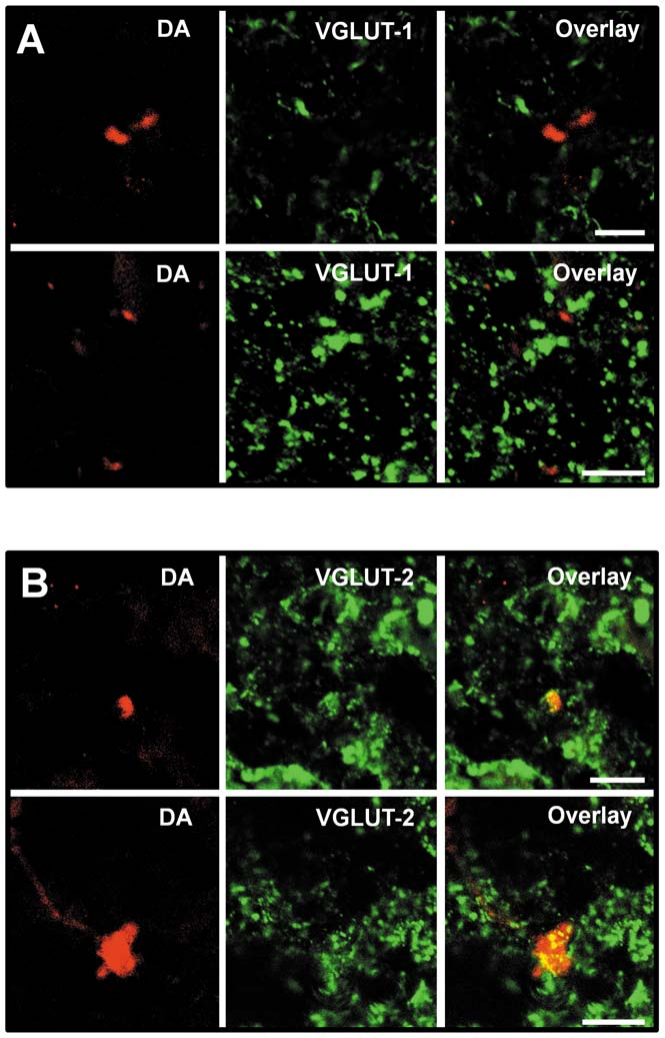
Projections from the LVN to the DCN do not co-label with VGLUT-1 and co-label with VGLUT-2. (**A**) Dextran amine (DA) labelled terminals are shown in the DCN deep layer (left). VGLUT-1 labelling is shown in the same area (middle). The overlay (right) shows an absence of co-labelling between VGLUT-1 and DA labelled terminals. (**B**) DA labelled terminals are shown in the DCN deep layer (left). VGLUT-2 labelling is shown in the same area (middle). The overlay (right) shows that the DA labelled terminals co-label with VGLUT-2. Scale bar 10 µm.

Four brainstems were injected with anterograde tracers in the ipsilateral LVN. The immunostaining procedure of VGLUT-1 and VGLUT-2 was performed within a month period (see methods). Labelled terminals originating from the LVN failed to co-localize with VGLUT-1 (Figure 6A) with only 0.5±0.4% (n = 267 terminals, N = 4) of the LVN terminal endings to the DCN co-labeled with VGLUT-1. By contrast the majority of terminals originating from the LVN co-localized with VGLUT-2 (Figure 6B, 85%±3.6%, n = 209 terminals, N = 4). VGLUT-2 labelled terminals originating from the LVN were found in both deep and fusiform layers (54.5% and 45.5% respectively) and consisted of labelled mossy fiber terminals (diameter of 2.7±0.7 µm, 41.5%, n = 70 terminals in 18 fields, N = 4) and small boutons (diameter of 1.4±0.4 µm, 58.5%, n = 80 terminals in 18 fields, N = 4).VGLUT-2 labelled LVN terminals did not show morphological or topographical differences from those that did not co-label with VGLUT-1 or VGLUT-2.

### Expression of VGLUT-2 labelled terminals from the lateral vestibular nucleus is increased after acoustic overexposure (AOE)

**Figure 7:**
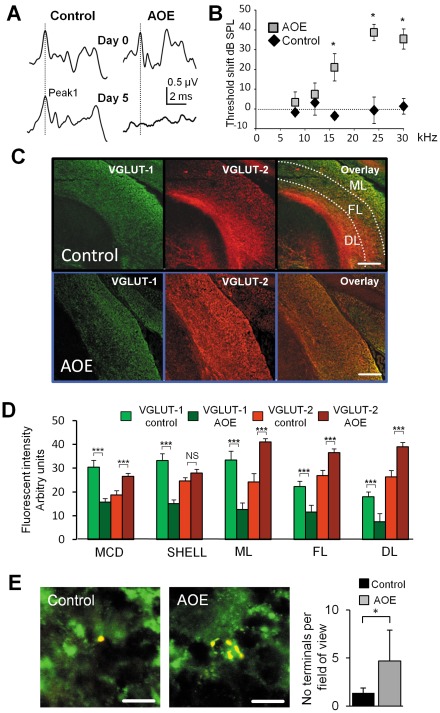
VGLUT-2 immunoreactivity increases after acoustic overexposure (AOE) triggering hearing deficit. (**A**) Auditory brainstem response (ABR) recordings are elicited by a tone pip of 24 kHz and 94 dB SPL. The top traces show ABRs obtained at day 0 in a control subject and prior to AOE. The bottom traces show ABRs obtained at day 5 in both subjects. After AOE, the ABR is characterised by a flat trace and wave I (shown by the dotted line) is absent. (**B**) Summary plot representing the ABR threshold shifts between day 0 and day 5 in response to the tone pip frequency. * p<0.05. (**C**) Expression of VGLUT-1 (left) and VGLUT-2 (middle) in DCN coronal slices originating from a control subject (top) and after AOE (bottom). The overlay of VGLUT-1 and VGLUT-2 is shown in the right panels. (**D**) Histograms representing the fluorescence intensity of VGLUT-1 and VGLUT-2 in the DCN layers, the magnocellular domain of the VCN MCD and the shell region, *** P<0.001, NS non significant. ML: molecular layer; FL: fusiform cell layer; DL: deep layer. After AOE, the VGLUT-1 immunoreactivity decreases and the VGLUT-2 immunoreactivity increases in all DCN layers and the MCD. VGLUT-1 is decreased after AOE in the shell but VGLUT-2 expression was unaffected. (**E**) Examples of synaptic boutons originating from the LVN labelled with VGLUT-2 in control condition (left) and after AOE (middle). Note the presence of multiple VGLUT-2 labelled terminals after AOE. Results are summarised in the histogram (right). * p<0.05, Scale bar: (C) 100 µm, (E) 5 µm.

Wistar rats were subjected to a loud 15 kHz tone for 2 times 3 hours (AOE, N = 3, control, N = 3). After 5 days, this procedure affected the auditory brainstem responses (Figure 7A) and increased the hearing thresholds for frequencies of 16 kHz and above (N = 3, Figure 7B). AOE decreased the levels of intensity of VGLUT-1 in all areas studied (the MCD, the shell region as well as the molecular, fusiform and deep layers of the DCN and the granule cell domain, p<0.001, Mann Whitney test, n = 9 fields, N = 3 for controls and AOE, Figure 7C–D. By contrast AOE increased the levels of intensity of VGLUT-2 in the MCD as well as the molecular, fusiform and deep layers of the DCN (p<0.001, Mann Whitney test n = 9 fields, N = 3 for all subjects controls and AOE, Figure 7C–D). No significant change of the intensity of VGLUT-2 was observed in the shell region (p = 0.48, Mann Whitney test, n = 9 fields, N = 3, Figure 7C–D). When animals were previously exposed to loud sound (AOE), the number of terminals originating from the LVN (located in the deep and fusiform layers) and co-labeled with VGLUT-2 increased from 1.4±0.5 per 5000 µm^2^ area, (n = 18 fields, N = 3) to 4.7±3.2 per 5000 µm^2^ area (n = 19 fields, N = 3, P<0.001, unpaired t test). The percentage of VGLUT-2 labelled terminals originating from the LVN increased from 1.6±0.1% (n = 22 fields, N = 3) to 5.8±0.7% (n = 22 fields, N = 3) P<0.001, unpaired t test). Examples of dextran amine labelled small boutons and mossy fiber terminals can be seen in Figure 7E and Figure S3 respectively. The distribution of labelled terminals into the DCN remained similar after AOE (46.1% in the deep layer and 53.9% in the fusiform cell layer, n = 22 fields, N = 3) as did the proportion of the mossy fibers and small boutons (56.5%, n = 22 fields, N = 3 and 43.5%, n = 22, N = 3 respectively). Mossy fiber terminal sizes remained unaffected after AOE (2.8±0.6 µm n = 21 fields, N = 3 control and 2.7±0.5 µm, n = 21 fields, N = 3 AOE, P = 0.4 unpaired T test) as did the size of the small boutons (1.4±0.3 µm, n = 21 fields, N = 3 control and 1.2±0.3 µm, n = 22 fields, N = 3 AOE respectively, P = 0.4 unpaired T test). We tested whether the size of EPSCs recorded in fusiform cells was modulated after AOE. Seven days after AOE, EPSCs blocked by D-AP5 and NBQX were elicited by LVN stimulations in 3 out of 20 fusiform cells (N = 6), with a similar amplitude (60±31 pA, n = 3 cells) to control conditions (P = 0.72, unpaired T test). This shows that the increased expression of the LVN mediated VGLUT-2 terminals after AOE did not affect the size of the EPSCs.

## Discussion

### Description of synaptic projections from the lateral vestibular nucleus to the dorsal cochlear nucleus

So far, reported documentation of vestibular projections to the DCN remains scarce [23], [24]. This study has been able to further elucidate the connections between the vestibular nuclear complex showing a connection between the LVN and the DCN. Development of a combined focal electroporation and pressure ejection system [39] has enabled us to focally apply neuronal tracers precisely to brainstem nuclei and faithfully trace their projections. Retrograde transport of DA from the DCN showed labelling of cell bodies in the LVN, and the reciprocal anterograde tract-tracing experiment resulted in terminals labelled in the ispilateral DCN. It is of note that other nuclei project to the DCN [10], [16], [17], [49]. However many of these (such as the Raphe and cuneate nucleus) occur prior to the injection level and have therefore not been included in this study. Since injections in this study were entirely contained within the boundaries of the LVN, these anterograde projections are representative of the pathway from the LVN to the DCN. Whilst both labelled cell bodies and terminals were observed in both sagittal and coronal sections, the number labelled in corresponding coronal sections was visibly fewer. This may be due to axonal projections being cut prior to dextran amine injection in the coronal plane as axons might deviate around fiber tracts such as the inferior cerebellar peduncle. Terminal endings originating from the LVN consisted of small boutons or large and irregular mossy fibers that were previously described as containing numerous synaptic vesicles [50]. Both types of synaptic terminals were located in the granule cell domain of the fusiform and the deep layer of the DCN similar to other non auditory projections such as the cuneate and the spinal trigeminal nucleus that are also confined to those regions [13], [31]. The role of mossy fiber terminals and small boutons in the DCN remain poorly understood. In the hippocampus, very brief (sub millisecond) presynaptic spikes at mossy fibers [51] are conferred by a distinct set of voltage-gated ion channels to ensure the synchrony of transmitter release at those synapses. Mossy fibers in the DCN could probably carry fast excitatory inputs and allow temporal precision in granule cell areas whereas small boutons could be involved in a slower modulatory function. It is noticeable that projections originating in the cuneate and the spinal trigeminal nucleus and terminating in the DCN showed more small boutons than mossy fibers [13] whereas projections originating from the LVN showed an equal proportion of small boutons and mossy fiber terminals. The pathway-specific relative proportion of mossy fiber terminals and small boutons may therefore be associated with the intrinsic synaptic properties that are unique to each sensory pathway.

### Pattern of expression of VGLUT-1 and VGLUT-2 positive terminals

Our study shows that VGLUT-1 is strongly expressed in the MCD, the DCN molecular layer and the shell region. The MCD is primarily innervated by the axons of the type I spiral ganglion neurons [52] and our results are consistent with auditory nerve projections being associated with VGLUT-1. The fact that VGLUT-1 is also concentrated in the molecular layer also observed in [13], [31] could sound counter-intuitive since VGLUT-1 is associated with auditory nerve fibers which project to the MCD and deep layers of the DCN [31]. However it has been suggested that VGLUT-1 in the molecular layer could be associated with parallel fiber projections in the DCN [31] as cerebellar parallel fiber terminals also contain VGLUT-1 for accumulation of glutamate into the synaptic vesicles [25], [53]. VGLUT-1 in the DCN molecular layer could also be associated with descending inputs from higher auditory centres such as the auditory cortex with projections terminating in all three layers of the DCN including the molecular layer [54], [55]. The high density of VGLUT-1 labelling in the shell region is more surprising as the shell region does not receive inputs from myelinated nerve fibers [52] labelled with VGLUT-1 [31] but from unmyelinated type II fibers [56]. The shell region contains granule cells and a variety of other neuronal types with different morphologies [57] and it is possible that VGLUT-1 is expressed at specific synaptic regions. By contrast to VGLUT-1, VGLUT-2 is most strongly expressed in the DCN deep layer receiving both auditory and non auditory projections [13], [31]. Similarly to [31], we found that VGLUT-2 was also highly expressed in the fusiform cell layer receiving non auditory projections [11]. In summary, despite differences in the proportion of VGLUT-1 and VGLUT-2 reported in previous studies, our finding is mostly consistent with auditory and multi-sensory inputs to the DCN being associated with VGLUT-1 and VGLUT-2, respectively [31]. Differential expression of VGLUT-1 and VGLUT-2 might result in changes in vesicle filling and recycling [58], [59] and/or differing release probabilities at the synapse [53]. It was suggested that VGLUT-1 characterises terminals with a low release probability present in highly plastic synapses whereas terminals containing VGLUT-2 have a higher release probability [53]. Therefore the complementary expression of these two transporter proteins could help optimizing the properties of glutamatergic synapses localised in specific regions.

### Role of VGLUT-2 mediated projections from the lateral vestibular nucleus to the dorsal cochlear nucleus

The co-localization of VGLUT2 with LVN terminals in the DCN shown in this study confirms that the LVN pathway to the DCN is glutamatergic and demonstrates that projections from the LVN, like those from the spinal trigeminal nucleus and the cuneate nucleus [11], [13], use VGLUT-2 to mediate glutamate transport at both mossy fiber and small boutons terminal endings. Whether this translates into higher release probability synapses compared to VGLUT-1 mediated synapses in the DCN needs to be determined. We report that the projections from the LVN to DCN are functional, eliciting glutamatergic EPSCs in fusiform cells and granule interneurones. Previous reports suggested that the somatosensory projections into the DCN convey information regarding head, neck and ear position and assist sound localization [18], [20], [31], [60]. The role of the vestibular projections to the DCN remains unclear. The vestibular system senses the movement and position of the head in space to stabilise vision, control posture, detect head orientation and self-motion in three-dimensional space and modulate autonomic and limbic system activities in response to locomotion and postural adjustments [61]. It is possible that vestibular projections to the DCN provide information on head orientation with respect to the DCN encoding spectral components of sound [21]. Projections from the LVN to DCN represent 2% of the total number of VGLUT-2 labelled multisensory projections. This could explain the small percentage of DCN fusiform cells and granule cells that display an EPSC in response to stimulating the LVN. However the small percentage of EPSCs could also be due to cut axonal projections during the slicing procedure. As the LVN integrates the information of linear acceleration and gravity changes to control the vestibulo-spinal reflexes and posture [62], [63] its role might become apparent during postural adjustments and head positioning at the beginning of the locomotion [64], [65]. Projections from the LVN and the somatosensory system might therefore play complementary roles in the orientation of head towards sound.

### Expression modulation of VGLUT-2 labeled terminals from the lateral vestibular nucleus

Acoustic over exposure (AOE) can induce cochlear damage with resultant damage to the hair cells, spiral ganglion neurones and the myelin sheath of the auditory nerve [66], [67]. Indeed our observations show an increase in the hearing thresholds five days after initial AOE. Whilst previous studies have used kanamycin-induced deafness or cochlear ablation which both cause complete hearing loss [30], [34], [68], our study uses AOE as a physiological insult to induce partial hearing deficit and monitoring the subsequent impact on the regulation of VGLUT's. Following AOE, we observed a decrease in VGLUT-1 expression mainly in the VCN and the molecular layer of the DCN. This is in accordance with a previous study showing that VGLUT-1 expression changed in the VCN as early as 3 days following deafness [30]. Moreover VGLUT-1 immunostaining was no longer seen in large auditory nerve terminals but was instead found in somata of VCN neurons [30]. VGLUT-2 levels conversely increased across the DCN and the VCN. This concurs with previous patterns which describe increased VGLUT-2 expression in the regions of the DCN that receive non auditory inputs after kanamycin induced deafness [34]. Cochlear deafferentation results in a reduced synaptic input from the auditory nerve [69], [70] and an enhanced somatosensory response in the DCN after noise induced hearing loss [71].

An increase in the expression of VGLUT-2 could indicate a compensatory mechanism whereby specific synaptic inputs to the DCN are increased in response to the loss of synaptic activity from the auditory nerve. The inputs from the LVN would therefore consist of a compensatory mechanism originating from the vestibular system. Cross modal compensation has previously been demonstrated after deafferentation or sensory deprivation in other modalities [72], [73] although the majority of compensation has been observed in cortical structures [74]–[76]. Whilst acting as a possible compensatory mechanism, the increase in VGLUT-2 expression could also lead to an increased release in glutamate due to larger vesicles [77] or to axonal sprouting [78]. An increase of presynaptic release of glutamate could contribute to post synaptic neuronal damage [79] and/or to the hyperactivity in the DCN [71], [80]–[82] correlating with tinnitus [81], [83]–[85]. Whilst juvenile rats were used in this study it is worth noting that hearing loss at an early age can cause a much more severe impairment on neural plasticity [86], audiogenic seizures [87] and acoustic startle reflex [88]. However changes in activity and VGLUT-2 up-regulation have also been reported in adult animals [34], [89], [90] and it is therefore possible that VGLUT-2 up-regulation as a result of AOE would also occur in adult animals. Although we did not observe an increase of the size of the EPSCs after AOE, this could be to due to the sparseness of functional connections in slices containing the LVN and the DCN. However the release of glutamate is dependent on an independent regulation of synaptic vesicle protein trafficking and recycling [91], and it is possible that glutamate release is still unaffected at those early stages after AOE. Another possibility could be that it is only the recycling rate that is affected. For example, VGLUT-2 mediated transmission at hippocampal synapses depress more rapidly and recover more slowly than VGLUT-1 mediated synaptic transmission [59].

It is of interest that vestibular conditions such as vertigo or vestibular schwannoma can result in tinnitus [92] and successful treatment of these vestibular conditions results in an abolishment of tinnitus [93]. Many tinnitus sufferers are also able to modulate the tinnitus percepts with head or neck manipulations [94]–[96]. It is possible that the projections from the LVN to the DCN contribute to the pathophysiology of tinnitus in relation to vestibular schwannoma and/or to the modulatory effect of tinnitus by head and neck manipulations.

## Supporting Information

Figure S1 **Sagittal (top) and coronal (bottom) brainstem slice showing the site of injection of dextran amine in the dorsal cochlear nucleus DCN.** Left, overlays of the brightfield and fluorescence photomicrographs at 3 hours post injection of dextran amine. Images on the right show the fluorescent micrographs. Scale bar 100 µm.
(TIF)

Figure S2 **High magnification photomicrographs showing VGLUT-1 and VGLUT-2 positive puncta in the MCD (A), the shell region (B) and the layers of the DCN (C–E).** VGLUT-1 positive puncta are most densely located in the MCD and molecular layer of the DCN (A and C) whereas VGLUT-2 puncta show a greater density than VGLUT-1 in the shell region, the fusiform and deep layers (B, D and E). Scale bar 2 µm.
(TIF)

Figure S3 **Examples of mossy fiber terminals originating from the LVN labelled with VGLUT-2 in control condition (left) and after AOE (middle).** Note the presence of multiple VGLUT-2 labelled mossy fiber terminals after AOE. Scale bar 5 µm.
(TIF)

## References

[1] Browner RH, Webster DB (1975). Projections of the trapezoid body and the superior olivary complex of the Kangaroo rat (Dipodomys merriami).. Brain Behav Evol.

[2] Adams JC (1983). Multipolar cells in the ventral cochlear nucleus project to the dorsal cochlear nucleus and the inferior colliculus.. Neurosci Lett.

[3] Snyder RL, Leake PA (1988). Intrinsic connections within and between cochlear nucleus subdivisions in cat.. J Comp Neurol.

[4] Doucet JR, Ryugo DK (1997). Projections from the ventral cochlear nucleus to the dorsal cochlear nucleus in rats.. J Comp Neurol.

[5] Babalian AL, Ryugo DK, Rouiller EM (2003). Discharge properties of identified cochlear nucleus neurons and auditory nerve fibers in response to repetitive electrical stimulation of the auditory nerve.. Exp Brain Res.

[6] Bukowska D, Zguczynski L (2003). Auditory centre projection of the lower brainstem to the dorsal cochlear nucleus in the rabbit.. Arch Ital Biol.

[7] Sutherland DP, Masterton RB, Glendenning KK (1998). Role of acoustic striae in hearing: reflexive responses to elevated sound-sources.. Behav Brain Res.

[8] May BJ (2000). Role of the dorsal cochlear nucleus in the sound localization behavior of cats.. Hear Res.

[9] Shore SE (2005). Multisensory integration in the dorsal cochlear nucleus: unit responses to acoustic and trigeminal ganglion stimulation.. Eur J Neurosci.

[10] Shore SE, Vass Z, Wys NL, Altschuler RA (2000). Trigeminal ganglion innervates the auditory brainstem.. J Comp Neurol.

[11] Zhou J, Shore S (2004). Projections from the trigeminal nuclear complex to the cochlear nuclei: a retrograde and anterograde tracing study in the guinea pig.. J Neurosci Res.

[12] Koehler SD, Pradhan S, Manis PB, Shore SE (2011). Somatosensory inputs modify auditory spike timing in dorsal cochlear nucleus principal cells.. Eur J Neurosci.

[13] Zeng C, Shroff H, Shore SE (2011). Cuneate and spinal trigeminal nucleus projections to the cochlear nucleus are differentially associated with vesicular glutamate transporter-2.. Neuroscience.

[14] Babalian AL (2005). Synaptic influences of pontine nuclei on cochlear nucleus cells.. Exp Brain Res.

[15] Itoh K, Kamiya H, Mitani A, Yasui Y, Takada M (1987). Direct projections from the dorsal column nuclei and the spinal trigeminal nuclei to the cochlear nuclei in the cat.. Brain Res.

[16] Weinberg RJ, Rustioni A (1987). A cuneocochlear pathway in the rat.. Neuroscience.

[17] Weinberg RJ, Rustioni A (1989). Brainstem projections to the rat cuneate nucleus.. J Comp Neurol.

[18] Young ED, Nelken I, Conley RA (1995). Somatosensory effects on neurons in dorsal cochlear nucleus.. J Neurophysiol.

[19] Thompson AM, Moore KR, Thompson GC (1995). Distribution and origin of serotoninergic afferents to guinea pig cochlear nucleus.. J Comp Neurol.

[20] Kanold PO, Young ED (2001). Proprioceptive information from the pinna provides somatosensory input to cat dorsal cochlear nucleus.. J Neurosci.

[21] Oertel D, Young ED (2004). What's a cerebellar circuit doing in the auditory system?. Trends Neurosci.

[22] Shore SE, Zhou J (2006). Somatosensory influence on the cochlear nucleus and beyond.. Hear Res.

[23] Burian M, Gstoettner W (1988). Projection of primary vestibular afferent fibres to the cochlear nucleus in the guinea pig.. Neurosci Lett.

[24] Bukowska D (2002). Morphological evidence for secondary vestibular afferent connections to the dorsal cochlear nucleus in the rabbit.. Cells Tissues Organs.

[25] Kaneko T, Fujiyama F (2002). Complementary distribution of vesicular glutamate transporters in the central nervous system.. Neurosci Res.

[26] Blaesse P, Ehrhardt S, Friauf E, Nothwang HG (2005). Developmental pattern of three vesicular glutamate transporters in the rat superior olivary complex.. Cell Tissue Res.

[27] Billups B (2005). Colocalization of vesicular glutamate transporters in the rat superior olivary complex.. Neurosci Lett.

[28] Ito T, Bishop DC, Oliver DL (2011). Expression of glutamate and inhibitory amino acid vesicular transporters in the rodent auditory brainstem.. J Comp Neurol.

[29] Herzog E, Gilchrist J, Gras C, Muzerelle A, Ravassard P (2004). Localization of VGLUT3, the vesicular glutamate transporter type 3, in the rat brain.. Neuroscience.

[30] Fyk-Kolodziej B, Shimano T, Gong TW, Holt AG (2011). Vesicular glutamate transporters: spatio-temporal plasticity following hearing loss.. Neuroscience.

[31] Zhou J, Nannapaneni N, Shore S (2007). Vessicular glutamate transporters 1 and 2 are differentially associated with auditory nerve and spinal trigeminal inputs to the cochlear nucleus.. J Comp Neurol.

[32] Tascioglu AB (2005). Brief review of vestibular system anatomy and its higher order projections.. Neuroanatomy.

[33] Zhang FX, Pang YW, Zhang MM, Zhang T, Dong YL (2011). Expression of vesicular glutamate transporters in peripheral vestibular structures and vestibular nuclear complex of rat.. Neuroscience.

[34] Zeng C, Nannapaneni N, Zhou J, Hughes LF, Shore S (2009). Cochlear damage changes the distribution of vesicular glutamate transporters associated with auditory and nonauditory inputs to the cochlear nucleus.. J Neurosci.

[35] Morest DK, Bohne BA (1983). Noise-induced degeneration in the brain and representation of inner and outer hair cells.. Hear Res.

[36] Kim J, Morest DK, Bohne BA (1997). Degeneration of axons in the brainstem of the chinchilla after auditory overstimulation.. Hear Res.

[37] Morest DK, Kim J, Potashner SJ, Bohne BA (1998). Long-term degeneration in the cochlear nerve and cochlear nucleus of the adult chinchilla following acoustic overstimulation.. Microsc Res Tech.

[38] Paxinos G, Watson C (2007). The rat brain..

[39] Barker M, Billups B, Hamann M (2009). Focal macromolecule delivery in neuronal tissue using simultaneous pressure ejection and local electroporation.. J Neurosci Methods.

[40] Freeman S, Geal-Dor M, Sohmer H (1999). Development of inner ear (cochlear and vestibular) function in the fetus-neonate.. J Basic Clin Physiol Pharmacol.

[41] Murashita H, Tabuchi K, Hoshino T, Tsuji S, Hara A (2006). The effects of tempol, 3-aminobenzamide and nitric oxide synthase inhibitors on acoustic injury of the mouse cochlea.. Hear Res.

[42] Webster DB, Trune DR (1982). Cochlear nuclear complex of mice.. Am J Anat.

[43] Hackney CM, Osen KK, Kolston J (1990). Anatomy of the cochlear nuclear complex of guinea pig.. Anat Embryol (Berl).

[44] Pilati N, Barker M, Panteleimonitis S, Donga R, Hamann M (2008). A rapid method combining Golgi and Nissl staining to study neuronal morphology and cytoarchitecture.. J Histochem Cytochem.

[45] Zeng C, Shroff H, Shore SE (2011). Cuneate and spinal trigeminal nucleus projections to the cochlear nucleus are differentially associated with vesicular glutamate transporter-2.. Neuroscience.

[46] Kanold PO, Manis PB (1999). Transient potassium currents regulate the discharge patterns of dorsal cochlear nucleus pyramidal cells.. J Neurosci.

[47] Street SE, Manis PB (2007). Action potential timing precision in dorsal cochlear nucleus pyramidal cells.. J Neurophysiol.

[48] Balakrishnan V, Trussell LO (2008). Synaptic inputs to granule cells of the dorsal cochlear nucleus.. J Neurophysiol.

[49] Wright DD, Ryugo DK (1996). Mossy fiber projections from the cuneate nucleus to the cochlear nucleus in the rat.. J Comp Neurol.

[50] Mugnaini E, Warr WB, Osen KK (1980). Distribution and light microscopic features of granule cells in the cochlear nuclei of cat, rat, and mouse.. J Comp Neurol.

[51] Bischofberger J, Engel D, Li L, Geiger JR, Jonas P (2006). Patch-clamp recording from mossy fiber terminals in hippocampal slices.. Nat Protoc.

[52] Fekete DM, Rouiller EM, Liberman MC, Ryugo DK (1984). The central projections of intracellularly labeled auditory nerve fibers in cats.. J Comp Neurol.

[53] Fremeau RT, Troyer MD, Pahner I, Nygaard GO, Tran CH (2001). The expression of vesicular glutamate transporters defines two classes of excitatory synapse.. Neuron.

[54] Jacomme AV, Nodal FR, Bajo VM, Manunta Y, Edeline JM (2003). The projection from auditory cortex to cochlear nucleus in guinea pigs: an in vivo anatomical and in vitro electrophysiological study.. Exp Brain Res.

[55] Schofield BR, Coomes DL (2005). Projections from auditory cortex contact cells in the cochlear nucleus that project to the inferior colliculus.. Hear Res.

[56] Brown MC, Ledwith JV (1990). Projections of thin (type-II) and thick (type-I) auditory-nerve fibers into the cochlear nucleus of the mouse.. Hear Res.

[57] Ryugo DK, Haenggeli CA, Doucet JR (2003). Multimodal inputs to the granule cell domain of the cochlear nucleus.. Exp Brain Res.

[58] Herzog E, Bellenchi GC, Gras C, Bernard V, Ravassard P (2001). The existence of a second vesicular glutamate transporter specifies subpopulations of glutamatergic neurons.. J Neurosci.

[59] Fremeau RT, Voglmaier S, Seal RP, Edwards RH (2004). VGLUTs define subsets of excitatory neurons and suggest novel roles for glutamate.. Trends Neurosci.

[60] Davis KA, Miller RL, Young ED (1996). Effects of somatosensory and parallel-fiber stimulation on neurons in dorsal cochlear nucleus.. J Neurophysiol.

[61] Angelaki DE, Cullen KE (2008). Vestibular system: the many facets of a multimodal sense.. Annu Rev Neurosci.

[62] Chan YS, Hwang JC, Cheung YM (1979). Vestibular function of saccule in cats as indicated by the response of Deiters' nucleus to static tilts.. Exp Brain Res.

[63] Lai SK, Lai CH, Yung KK, Shum DK, Chan YS (2006). Maturation of otolith-related brainstem neurons in the detection of vertical linear acceleration in rats.. Eur J Neurosci.

[64] Orlovsky GN (1972). Activity of vestibulospinal neurons during locomotion.. Brain Res.

[65] Marlinsky VV (1992). Activity of lateral vestibular nucleus neurons during locomotion in the decerebrate guinea pig.. Exp Brain Res.

[66] Lawner BE, Harding GW, Bohne BA (1997). Time course of nerve-fiber regeneration in the noise-damaged mammalian cochlea.. Int J Dev Neurosci.

[67] Hurley PA, Crook JM, Shepherd RK (2007). Schwann cells revert to non-myelinating phenotypes in the deafened rat cochlea.. Eur J Neurosci.

[68] Kong WJ, Yin ZD, Fan GR, Li D, Huang X (2010). Time sequence of auditory nerve and spiral ganglion cell degeneration following chronic kanamycin-induced deafness in the guinea pig.. Brain Res.

[69] Muly SM, Gross JS, Morest DK, Potashner SJ (2002). Synaptophysin in the cochlear nucleus following acoustic trauma.. Exp Neurol.

[70] D'Sa C, Gross J, Francone VP, Morest DK (2007). Plasticity of synaptic endings in the cochlear nucleus following noise-induced hearing loss is facilitated in the adult FGF2 overexpressor mouse.. Eur J Neurosci.

[71] Shore SE, Koehler S, Oldakowski M, Hughes LF, Syed S (2008). Dorsal cochlear nucleus responses to somatosensory stimulation are enhanced after noise-induced hearing loss.. Eur J Neurosci.

[72] Goel A, Jiang B, Xu LW, Song L, Kirkwood A (2006). Cross-modal regulation of synaptic AMPA receptors in primary sensory cortices by visual experience.. Nat Neurosci.

[73] Oh SH, Kim CS, Song JJ (2007). Gene expression and plasticity in the rat auditory cortex after bilateral cochlear ablation.. Acta Otolaryngol.

[74] Batzri-Izraeli R, Kelly JB, Glendenning KK, Masterton RB, Wollberg Z (1990). Auditory cortex of the long-eared hedgehog (Hemiechinus auritus). I. Boundaries and frequency representation.. Brain Behav Evol.

[75] Izraeli R, Koay G, Lamish M, Heicklen-Klein AJ, Heffner HE (2002). Cross-modal neuroplasticity in neonatally enucleated hamsters: structure, electrophysiology and behaviour.. Eur J Neurosci.

[76] Piche M, Chabot N, Bronchti G, Miceli D, Lepore F (2007). Auditory responses in the visual cortex of neonatally enucleated rats.. Neuroscience.

[77] Daniels RW, Collins CA, Gelfand MV, Dant J, Brooks ES (2004). Increased expression of the Drosophila vesicular glutamate transporter leads to excess glutamate release and a compensatory decrease in quantal content.. J Neurosci.

[78] Kim JJ, Gross J, Morest DK, Potashner SJ (2004). Quantitative study of degeneration and new growth of axons and synaptic endings in the chinchilla cochlear nucleus after acoustic overstimulation.. J Neurosci Res.

[79] Daniels RW, Miller BR, DiAntonio A (2011). Increased vesicular glutamate transporter expression causes excitotoxic neurodegeneration.. Neurobiol Dis.

[80] Kaltenbach JA, Afman CE (2000). Hyperactivity in the dorsal cochlear nucleus after intense sound exposure and its resemblance to tone-evoked activity: a physiological model for tinnitus.. Hear Res.

[81] Brozoski TJ, Bauer CA, Caspary DM (2002). Elevated fusiform cell activity in the dorsal cochlear nucleus of chinchillas with psychophysical evidence of tinnitus.. J Neurosci.

[82] Sumner CJ, Tucci DL, Shore SE (2005). Responses of ventral cochlear nucleus neurons to contralateral sound after conductive hearing loss.. J Neurophysiol.

[83] Zhang JS, Kaltenbach JA (1998). Increases in spontaneous activity in the dorsal cochlear nucleus of the rat following exposure to high-intensity sound.. Neurosci Lett.

[84] Kaltenbach JA, Zacharek MA, Zhang J, Frederick S (2004). Activity in the dorsal cochlear nucleus of hamsters previously tested for tinnitus following intense tone exposure.. Neurosci Lett.

[85] Kaltenbach JA, Zhang J (2007). Intense sound-induced plasticity in the dorsal cochlear nucleus of rats: evidence for cholinergic receptor upregulation.. Hear Res.

[86] Sanes DH, Kotak VC (2011). Developmental plasticity of auditory cortical inhibitory synapses..

[87] Pierson M, Liebmann SL (1992). Noise exposure-induced audiogenic seizure susceptibility in Sprague-Dawley rats.. Epilepsy Res.

[88] Rybalko N, Bures Z, Burianova J, Popelar J, Grecova J (2011). Noise exposure during early development influences the acoustic startle reflex in adult rats.. Physiol Behav.

[89] Ding J, Benson TE, Voigt HF (1999). Acoustic and current-pulse responses of identified neurons in the dorsal cochlear nucleus of unanesthetized, decerebrate gerbils.. J Neurophysiol.

[90] Finlayson PG, Kaltenbach JA (2009). Alterations in the spontaneous discharge patterns of single units in the dorsal cochlear nucleus following intense sound exposure.. Hear Res.

[91] Santos MS, Li H, Voglmaier SM (2009). Synaptic vesicle protein trafficking at the glutamate synapse.. Neuroscience.

[92] Baguley DM, Humphriss RL, Axon PR, Moffat DA (2006). The clinical characteristics of tinnitus in patients with vestibular schwannoma.. Skull Base.

[93] Kameda K, Shono T, Hashiguchi K, Yoshida F, Sasaki T (2010). Effect of tumor removal on tinnitus in patients with vestibular schwannoma.. J Neurosurg.

[94] Herraiz C, Toledano A, Diges I (2007). Trans-electrical nerve stimulation (TENS) for somatic tinnitus.. Prog Brain Res.

[95] Levine RA (1999). Somatic (craniocervical) tinnitus and the dorsal cochlear nucleus hypothesis.. Am J Otolaryngol.

[96] Pinchoff RJ, Burkard RF, Salvi RJ, Coad ML, Lockwood AH (1998). Modulation of tinnitus by voluntary jaw movements.. Am J Otol.

