# Supplementary figures and images for: Acoustic Overexposure Increases the Expression of VGLUT-2 Mediated Projections from the Lateral Vestibular Nucleus to the Dorsal Cochlear Nucleus
Source: PLoS One. 2012 May 3;7(5):e35955. doi: 10.1371/journal.pone.0035955 (PMC3343051; doi:10.1371/journal.pone.0035955)

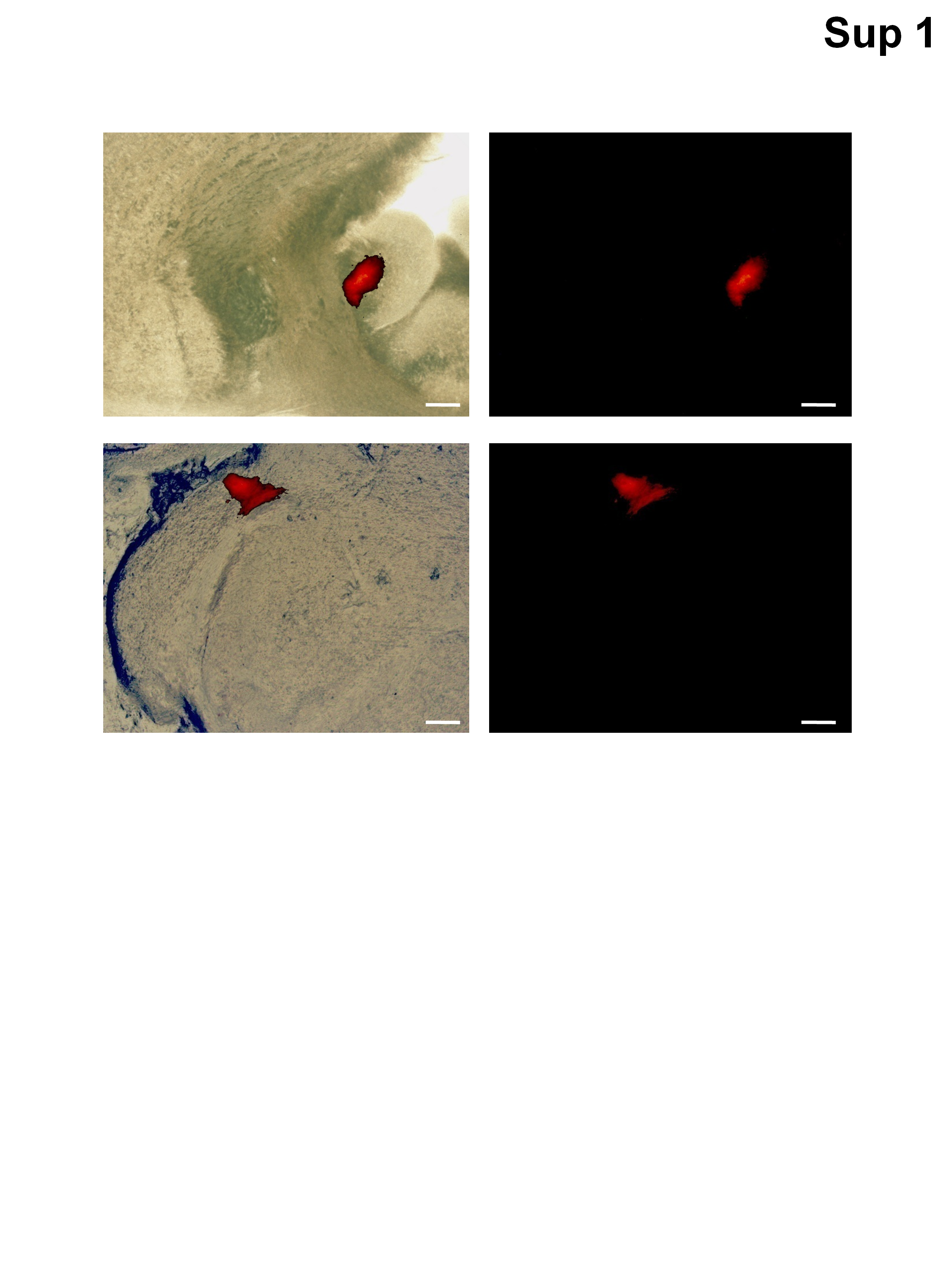

Supplement: Figure S1 — Sagittal (top) and coronal (bottom) brainstem slice showing the site of injection of dextran amine in the dorsal cochlear nucleus DCN. Left, overlays of the brightfield and fluorescence photomicrographs at 3 hours post injection of dextran amine. Images on the right show the fluorescent micrographs. Scale bar 100 µm. (TIF) [file pone.0035955.s001.tif]

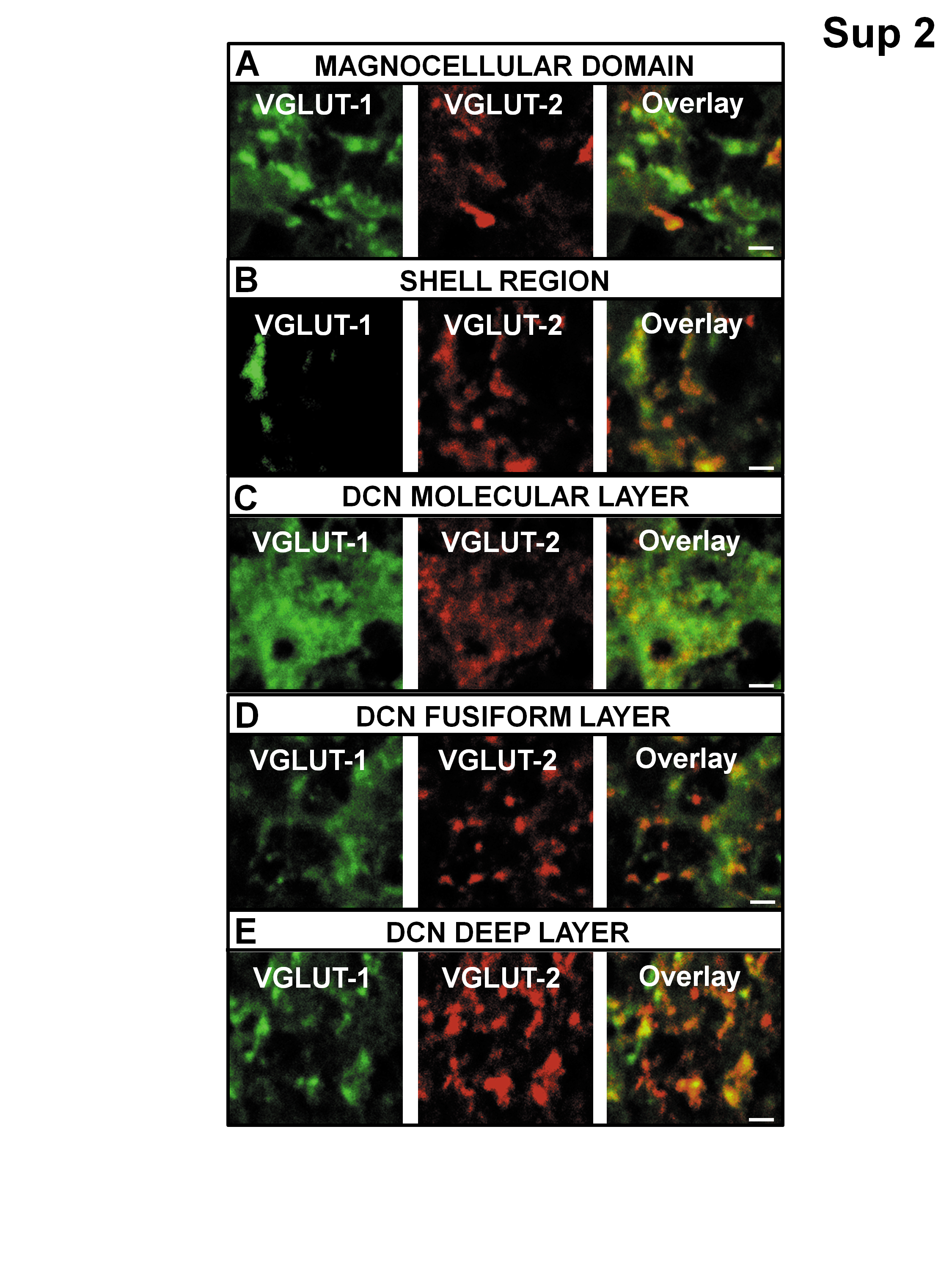

Supplement: Figure S2 — High magnification photomicrographs showing VGLUT-1 and VGLUT-2 positive puncta in the MCD (A), the shell region (B) and the layers of the DCN (C–E). VGLUT-1 positive puncta are most densely located in the MCD and molecular layer of the DCN (A and C) whereas VGLUT-2 puncta show a greater density than VGLUT-1 in the shell region, the fusiform and deep layers (B, D and E). Scale bar 2 µm. (TIF) [file pone.0035955.s002.tif]

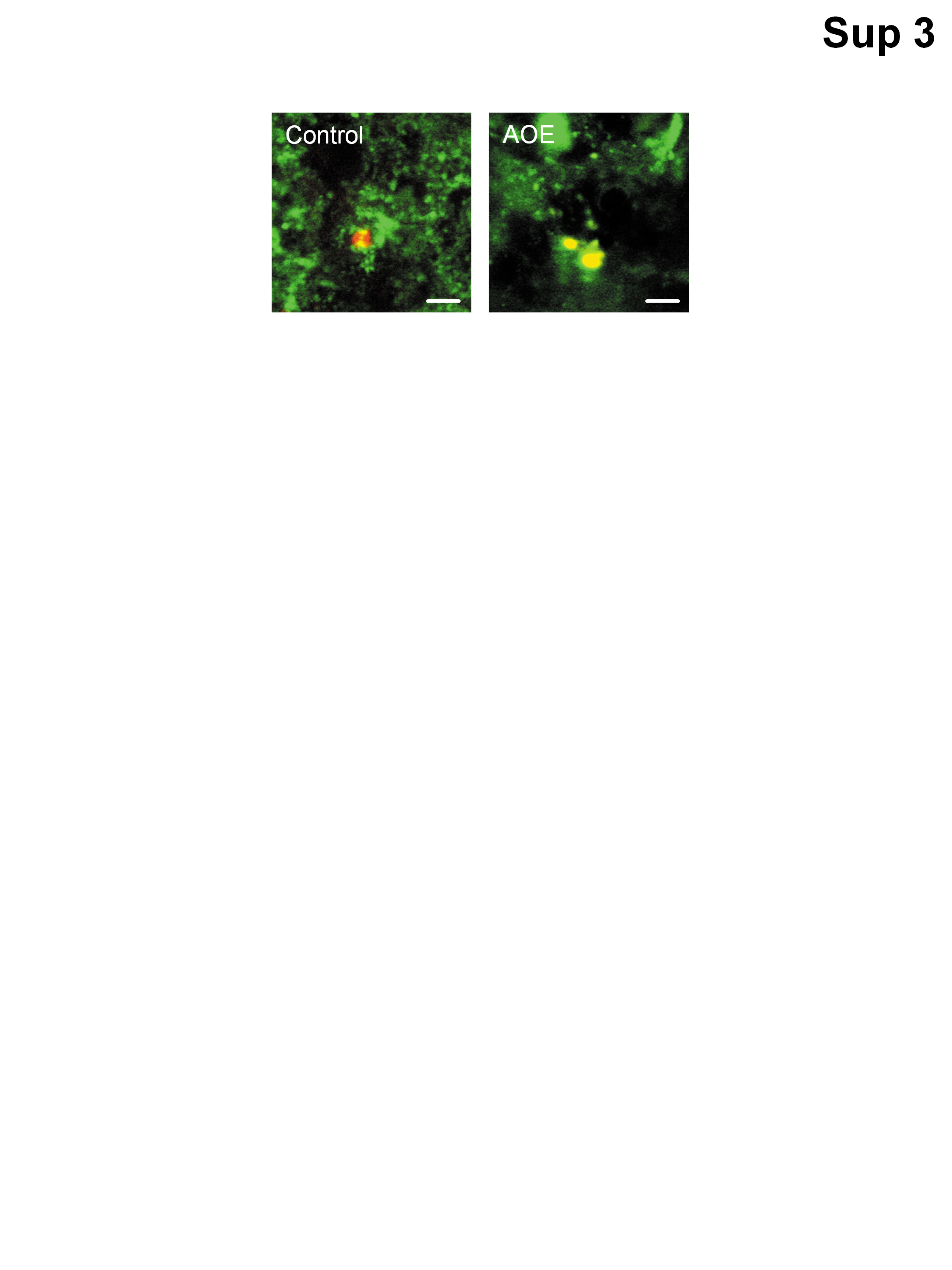

Supplement: Figure S3 — Examples of mossy fiber terminals originating from the LVN labelled with VGLUT-2 in control condition (left) and after AOE (middle). Note the presence of multiple VGLUT-2 labelled mossy fiber terminals after AOE. Scale bar 5 µm. (TIF) [file pone.0035955.s003.tif]
